# Socio-demographic determinants of *Toxoplasma gondii* seroprevalence in migrant workers of Peninsular Malaysia

**DOI:** 10.1186/s13071-017-2167-8

**Published:** 2017-05-15

**Authors:** Norhidayu Sahimin, Yvonne A. L. Lim, Farnaza Ariffin, Jerzy M. Behnke, Maria-Gloria Basáñez, Martin Walker, John W. Lewis, Rahmah Noordin, Khairul Anuar Abdullah, Siti Nursheena Mohd Zain

**Affiliations:** 1 Institute of Biological Science, Faculty of Science, University of Malaya, Kuala Lumpur, Malaysia; 2 Department of Parasitology, Faculty of Medicine, University of Malaya, Kuala Lumpur, Malaysia; 3 Primary Care Medicine, Faculty of Medicine, UiTM Sungai Buloh Campus, Sungai Buloh, Selangor Malaysia; 4 School of Life Sciences, University of Nottingham, University Park, Nottingham, NG7 2RD UK; 5 Department of Infectious Disease Epidemiology and London Centre for Neglected Tropical Disease Research, School of Public Health, St Mary’s Campus, Imperial College London, London, W2 1PG UK; 6 Department of Pathobiology and Population Sciences and London Centre for Neglected Tropical Disease Research, Royal Veterinary College, Hawkshead Lane, Hatfield, Hertfordshire AL97TA UK; 7 School of Biological Sciences, Royal Holloway, University of London, Egham, Surrey TW20 0EX UK; 8 Institute for Research Molecular Medicine, University of Science Malaysia, Gelugor, Penang Malaysia; 9 MAHSA University, Jalan Elmu off Jalan University, Kuala Lumpur, Malaysia

**Keywords:** *Toxoplasma gondii*, Migrant workers, Seroprevalence, Socio-demographic factors

## Abstract

**Background:**

The number of migrants working in Malaysia has increased sharply since the 1970’s and there is concern that infectious diseases endemic in other (e.g. neighbouring) countries may be inadvertently imported. Compulsory medical screening prior to entering the workforce does not include parasitic infections such as toxoplasmosis. Therefore, this study aimed to evaluate the seroprevalence of *T. gondii* infection among migrant workers in Peninsular Malaysia by means of serosurveys conducted on a voluntary basis among low-skilled and semi-skilled workers from five working sectors, namely, manufacturing, food service, agriculture and plantation, construction and domestic work.

**Methods:**

A total of 484 migrant workers originating from rural locations in neighbouring countries, namely, Indonesia (*n* = 247, 51.0%), Nepal (*n* = 99, 20.5%), Bangladesh (*n* = 72, 14.9%), India (*n* = 52, 10.7%) and Myanmar (*n* = 14, 2.9%) were included in this study.

**Results:**

The overall seroprevalence of *T. gondii* was 57.4% (*n* = 278; 95% CI: 52.7–61.8%) with 52.9% (*n* = 256; 95% CI: 48.4–57.2%) seropositive for anti-*Toxoplasma* IgG only, 0.8% (*n* = 4; 95% CI: 0.2–1.7%) seropositive for anti-*Toxoplasma* IgM only and 3.7% (*n* = 18; 95% CI: 2.1–5.4%) seropositive with both IgG and IgM antibodies. All positive samples with both IgG and IgM antibodies showed high avidity (> 40%), suggesting latent infection. Age (being older than 45 years), Nepalese nationality, manufacturing occupation, and being a newcomer in Malaysia (excepting domestic work) were positively and statistically significantly associated with seroprevalence (*P* < 0.05).

**Conclusions:**

The results of this study suggest that better promotion of knowledge about parasite transmission is required for both migrant workers and permanent residents in Malaysia. Efforts should be made to encourage improved personal hygiene before consumption of food and fluids, thorough cooking of meat and better disposal of feline excreta from domestic pets.

## Background

*Toxoplasma gondii* is one of the most common protozoan parasites affecting up to one-third of the world’s population [1–3]. Human infection may occur via ingestion of food or water contaminated with oocysts shed in the faeces of infected cats; consumption of undercooked or raw meat; consumption of raw oysters, clams, or mussels containing tissue cysts [4–8]; exposure to contaminated soil through activities such as gardening or children playing in sandpits [9] and vertical transmission from mother to foetus [10, 11].

Toxoplasmosis in immunocompromised people may causes damage to the brain, eyes, or other organs and is associated with severe acute infection or with reactivation of past infection. Infections acquired during pregnancy may cause severe damage to the foetus [11]. In immunocompromised patients, reactivation of latent infection can cause life-threatening encephalitis [2, 12]. In recent years, there have been also many attempts to link toxoplasmosis with schizophrenia and other mental health problems (such as bipolar disorder) [13–15]. *Toxoplasma gondii* has emerged as a prime candidate when investigating the relationship between infectious agents and schizophrenia; some individuals with adult toxoplasmosis develop psychotic symptoms [13].

The standard method for diagnosis is through serological testing, based on the detection of *Toxoplasma*-specific immunoglobulin IgG and IgM antibodies in serum and this test is routinely implemented in many parts of the world [16–18]. The detection methods previously employed in Malaysia have included the indirect hemagglutination (IHA) test, Sabin Feldman dye test, indirect fluorescent antibody test [19] and the enzyme-linked immunosorbent assay (ELISA) [20].

Over the years, the economy in Malaysia has transformed into an emerging multi-sector economy and since the 1970s, its economic vigour has been facilitated largely by imported migrant workers. The number of migrant workers arriving in Malaysia from neighbouring countries has grown exponentially and there is concern that diseases endemic in their countries may be inadvertently imported [21], despite compulsory medical screening prior to entering the workforce in Malaysia. However, pre-employment screening does not currently include screening for the presence of most parasitic infections, including toxoplasmosis. The infection could have substantial public health implications with regard to the productivity of the migrant labour force and its contribution to the Malaysian economy and well-being.

Previous studies have presented a mixed picture on the seroprevalence of *T. gondi* infection among migrant compared to indigenous workers in Malaysia. Chan et al. [21] reported up to 42% (138/336) positive for specific IgG while twenty mainly Indonesian plantation workers and workers in detention camps (6%) were positive with IgM [21]. Chan et al. [22] also noted the high prevalence of *T. gondii* infection among local plantation workers (IgG: *n* = 89, 44.9%) compared to migrants (*n* = 171, 34.1%). However, there was no statistically significant difference in the prevalence of raised IgM between migrant workers (*n* = 26, 5.2%) and locals (*n* = 17, 8.6%). Amal et al. [23] reported a lower rate of raised specific IgG among workers (*n* = 16, 18.8%) from the Indian subcontinent compared to locals (*n* = 89, 44.9%) from the same plantation and detention camp. Similarly, another study also showed that just over a third (34.1%, 171/501) of migrant plantation workers and individuals in detention camps were IgG positive and 5.2% (26/501) were IgM positive, with the highest infection rate among Nepalese workers (46.2%) compared to other ethnic groups [24].

The current study was a component of a broader project aiming to assess the range and extent of parasitic infections brought into Malaysia by the migrant worker population. The study is motivated by the need to assess the health status of migrant workers originating from countries with low socioeconomic backgrounds, living in deprived environments with poor sanitation and low hygiene practices [25]. Here we report on seroprevalence of *T. gondii* among migrant workers in Malaysia and identify key factors associated with this infection.

## Methods

### Study population and sample collection

This study was carried out from September 2014 to August 2015 among informed, consenting low-skilled and semi-skilled workers from five working sectors in Peninsular Malaysia, namely: manufacturing; service; agriculture and plantation; construction, and domestic work. Questionnaires were distributed to participants to gather relevant information related to the study. An individual clinical interview with a questionnaire was conducted to collect information on sociodemographic data, migration history, environmental health, life-style habits (consumption of raw meat and vegetables), recent illness and occupational health and safety. The interview process was performed through an interpreter for those migrant workers who had difficulty understanding the Malay (national) and English languages. All participants were fully informed of the nature of the study and completed the consent forms.

After consent was obtained and the questionnaire answered, approximately 5 ml of venous blood was drawn into a plain tube (without anticoagulant) by trained medical assistants and nurses using disposable syringes and needles. The blood samples were transported to the Parasitology Laboratory, Institute of Biological Science, Faculty of Science, University of Malaya. Blood samples were spun at 1,500× *g* for 10 min and the serum samples were kept in -20 °C until use.

### Detection of immunoglobulin G and M antibodies to *T. gondii*

Screening for anti-*T. gondii* antibodies was performed using enzyme-linked immunosorbent assay (ELISA) commercial kits for immunoglobulin G (IgG) and M (IgM) (Trinity Biotech Captia^TM^, New York, USA) in accordance with the manufacturer’s instructions. For the IgG assay, positive results were defined as ≥ 1.23 IU/ml, indicating latent or pre-existing *Toxoplasma* infection. Positive results for IgM assays were also defined as ≥ 1.23 IU/ml, indicating recent infection. All samples that were both IgG-positive and IgM-positive were tested using an IgG avidity assay (IgG; NovaLisa, Dietzenbach, Germany) according to the manufacturer’s instructions. *Toxoplasma* antibodies with high avidity (> 40%) indicate latent infection, while *Toxoplasma* antibodies with low avidity (≤40%) indicate a probable acute or recent infection.

### Data analysis

Prevalence estimates (percentage of participants infected) are shown with 95% confidence intervals (CI) calculated using the method described by Rohlf & Sokal (1995) [26]. Prevalence was analyzed using maximum likelihood techniques based on log linear analysis of contingency tables using the software package SPSS (Version 22), in three steps. First, full factorial models were fitted including the following ‘intrinsic’ factors: sex (2 levels, males and females), age (5 age classes comprising those < 25 years old, 25–34 years old, 35–44 years old, 45–54 years old and those > 54 years), nationality (5 countries, Bangladesh, India, Indonesia, Myanmar and Nepal) and immune status, which was considered as a binary factor (presence/absence of anti-*Toxoplasma* antibodies). For each level of analysis in turn, beginning with the most complex model, involving all possible main effects and interactions, those combinations that did not contribute significantly to explaining variation in the data were eliminated in a stepwise fashion beginning with the highest-level interaction (backward selection procedure in SPSS). A minimum sufficient model was then obtained, for which the likelihood ratio of the chi-square (*χ* ^2^) statistic was not significant, indicating that the model was sufficient in explaining the data. The importance of each term (i.e. interactions involving infection) in the final model was assessed by the probability that its exclusion would alter the model significantly and those values relating to interactions that included presence/absence of infection-specific antibodies (as described above) are given in the text.

Models were then fitted including the following ‘extrinsic’ factors: employment sector (5 sectors: construction, manufacture, plantation, food service and domestic), years of residence in Malaysia (2 categories: less than 1 year (year 1) and more than 1 year (year 2)), accommodation (3 types: hostel, construction site and own/rented home) and education (4 levels: primary school, secondary school, university and no formal schooling) and infection. Finally, in a third step, models were fitted comprising only the intrinsic and extrinsic factors that had been found to be statistically significantly associated with infection status as measured by presence of IgG/IgM.

Where relevant, we also fitted in turn models with just each factor and infection status, in order to resolve/clarify complex interactions that could not be simplified by the backward selection procedure. We have also provided in the tables the probability values from these models.

## Results

### Socio-demographic characteristics

A total of 484 migrant workers in Malaysia were included in this study originating from rural areas in neighbouring countries, namely: Indonesia (*n* = 247, 51.0%); Nepal (*n* = 99, 20.5%); Bangladesh (*n* = 72, 14.9%); India (*n* = 52, 10.7%), and Myanmar (*n* = 14, 2.9%). Slightly over three quarters (*n* = 375, 77.5%) were men, and the rest (*n* = 109, 22.5%) women. Most were between the ages of 25 and 34 years (*n* = 183, 37.8%), followed by less than 25 years (*n* = 142, 29.3%), between 35 and 44 years (*n* = 111, 22.9%), between 45 and 54 years (*n* = 35, 7.2%) and greater than 54 years (*n* = 13, 2.7%). According to the working sectors, the majority of volunteers were from the food service sector (*n* = 115, 23.8%), followed by domestic (*n* = 106, 21.9%), plantation (*n* = 102, 21.1%), manufacturing (*n* = 93, 19.2%) and construction (*n* = 68, 14.0%) sectors. Most participants had at least a primary level of education (*n* = 228, 47.1%) followed by high school (*n* = 201, 41.5%) and higher (university) level (*n* = 10, 2.1%), while *n* = 45 participants (9.3%) had not received any formal education.

### Seroprevalence of *T. gondii*

The overall *T. gondii* seroprevalence among 484 migrant workers was 57.4% (*n* = 278; 95% CI: 52.7–61.8%) with 52.9% (*n* = 256; 95% CI: 48.4–57.2%) being seropositive for anti-*Toxoplasma* IgG only, 0.8% (*n* = 4; 95% CI: 0.2–1.7%) seropositive for anti-*Toxoplasma* IgM only and 3.7% (*n* = 18; 95% CI: 2.1–5.4%) seropositive for both IgG and IgM antibodies (Table 1). All positive samples with both IgG and IgM antibodies showed high avidity (> 40%), suggesting latent infection.

**Table 1:** Seroprevalence of IgG and IgM antibodies to *Toxoplasma gondii* among 484 migrant workers using ELISA

Antibodies	No. of seropositive	Seropositive (%)	95% CI (%)
IgG+	257	53.0	48.3–57.7
IgM+	4	0.8	0.2–1.8
IgG+ IgM+	18	3.7	2.1–5.6
Total	279	57.5	53.2–62.1

*Abbreviation*: *CI* confidence interval

### Intrinsic factors associated with the seroprevalence of *T. gondii* infections

Seropositivity of *T. gondii* was analysed statistically in relation to sociodemographic factors. In the minimum sufficient model identified by the backwards stepwise selection procedure that included sex, age and nationality, only age (*χ* ^2^ = 11.989, *df* = 4, *P* = 0.017) and nationality (*χ* ^2^ = 32.275, *df* = 4, *P* ≤ 0.001) were found to be statistically significantly associated with seropositivity for *T. gondii* IgG, independently (Table 2). Analyses of anti-*Toxoplasma* IgM and seropositivity based on a combination of both IgG and IgM antibodies, did not find any of the three intrinsic factors as statistically significantly affecting seroprevalence (Table 2).

**Table 2:** Seroprevalence of IgG and IgM antibodies to *Toxoplasma gondii* infections among migrant workers in Malaysia according to sex, age, nationality, employment sector, years of residence, accommodation and education

Factors		IgG +		IgM+		IgG+ IgM+	
		% (95% CI)	*P*-value^a^	% (95% CI)	*P*-value	% (95% CI)	*P*-value
Intrinsic factors							
Sex	Men (*n* = 375)	55.7 (50.5–60.8)	0.469	4.8 (2.9–7.5)	0.610	3.7 (2.1–6.2)	0.975
	Women (*n* = 109)	59.6 (49.8–68.9)		3.7 (1.0–9.1)		3.7 (1.0–9.1)	
Age class (years)*	< 25 (*n* = 142)	59.2 (50.6–67.3)	0.045	3.5 (1.2–8.0)	0.732	3.5 (1.2–8.0)	0.853
	25–34 (*n* = 183)	51.4 (43.9–58.8)		5.5 (2.7–9.8)		3.8 (1.6–7.7)	
	35–44 (*n* = 111)	54.1 (44.3–63.6)		4.5 (1.5–10.2)		3.6 (1.0–9.0)	
	45–54 (*n* = 35)	74.3 (56.7–87.5)		5.7 (0.7–19.2)		5.7 (0.7–19.2)	
	> 55 (*n* = 13)	76.9 (46.2–95.0)		0.0 (0.0–24.7)		0.0 (0.0–24.7)	
Country of nationality*	Indonesia (*n* = 247)	58.3 (51.9–64.5)	< 0.001	5.3 (2.8–8.8)	0.448	5.3 (2.8–8.8)	0.325
	Bangladesh (*n* = 72)	44.4 (32.7–56.6)		2.8 (0.3–9.7)		1.4 (0.0–7.5)	
	Myanmar (*n* = 14)	28.6 (8.4–58.1)		0.0 (0.0–23.2)		0.0 (0.0–23.2)	
	India (*n* = 52)	38.5 (25.3–53.0)		1.9 (0.0–10.3)		1.9 (0.0–10.3)	
	Nepal (*n* = 99)	74.7 (65.0–82.9)		6.1 (2.3–12.7)		3.0 (0.6–8.6)	
Extrinsic factors							
Employment sector*	Construction (*n* = 68)	61.8 (49.2–73.3)	< 0.001	5.9 (1.6–14.4)	0.417	5.9 (1.6–14.4)	0.400
	Manufacturing (*n* = 93)	74.2 (64.1–82.7)		4.3 (1.2–10.6)		2.2 (0.3–7.6)	
	Plantation (*n* = 102)	44.1 (34.3–54.3)		4.9 (1.6–11.1)		3.9 (1.1–9.7)	
	Food service (*n* = 115)	50.4 (41.0–59.9)		1.7 (0.2–6.1)		1.7 (0.2–6.1)	
	Domestic (*n* = 106)	56.6 (46.6–66.2)		6.6 (2.7–13.1)		5.7 (2.1–11.9)	
Years of residence*	< than 1 year (*n* = 180)	65.0 (57.6–71.9)	0.004	6.7 (3.5–11.4)	0.091	5.6 (2.7–10.0)	0.107
	> than 1 year (*n* = 304)	51.6 (45.9–57.4)		3.3 (1.6–6.0)		2.6 (1.1–5.1)	
Accommodation	Own/rent house (*n* = 133)	57.1 (48.3–65.7)	0.638	5.3 (2.1–10.5)	0.447	4.5 (1.7–9.6)	0.236
	Construction site (*n* = 53)	62.3 (47.9–75.2)		7.5 (2.1–18.2)		7.5 (2.1–18.2)	
	Hostel by employer (*n* = 298)	55.4 (49.5–61.1)		3.7 (1.9–6.5)		2.7 (1.2–5.2)	
Education	Primary (*n* = 228)	54.4 (47.7–61.0)	0.114	4.8 (2.4–8.5)	0.674	3.9 (1.8–7.4)	0.572
	Secondary (*n* = 201)	62.7 (55.6–69.4)		4.0 (1.7–7.7)		3.0 (1.1–6.4)	
	University (*n* = 10)	50.0 (18.7–81.3)		0.0 (0.0–30.8)		0.0 (0.0–30.8)	
	No formal schooling (*n* = 45)	42.2 (27.7–57.8)		6.7 (1.4–18.3)		6.7 (1.4–18.3)	

*P < 0.01

^a^ *P*-values are based on each factor being fitted alone with presence/absence of antibodies and reflect the statistical significance of the variable in improving the fit of the model to the data. The results of the multifactorial models are described in the text

*Abbreviation*: *CI* confidence interval

### Extrinsic factors associated with the seroprevalence of *T. gondii* infections

Of the four extrinsic factors considered (employment sectors, years of residence in Malaysia, type of accommodation and level of education), only two factors were found to be statistically significantly associated with seropositivity of anti-*Toxoplasma* IgG in models that only included each factor in turn with presence of *Toxoplasma* IgG, i.e. employment sector (*χ* ^2^ = 21.306, *df* = 4, *P* ≤ 0.001) and years of residence in Malaysia (*χ* ^2^ = 8.294, *df* = 1, *P* = 0.004) (Table 2). Seroprevalence was significantly and positively associated with those employed in the manufacturing industry and those recently arrived in Malaysia (the latter with the exception of domestic workers, in whom the converse negative association with years of employment was observed).

Finally, in a multifactorial model in which we fitted only the significant effects from the analyses of intrinsic and extrinsic factors (age, country of origin, years of residence and employment sector with *Toxoplasma* IgG seropositivity), three significant interactions were found, the strongest being employment sector interacting with years of residence and *Toxoplasma* IgG (*χ* ^2^ = 13.478, *df* = 4, *P* = 0.009). In four cases (employment sectors construction, manufacturing, plantation and the service industry), prevalence was higher in year 1 (72.7, 79.5, 48.6 and 41.7%, respectively) than in year 2 (59.6, 55.0, 41.8 and 43.6%, respectively), indicating a reduction in infection between the two years, whilst for those in the domestic sector, prevalence increased from 41.7% in year 1 to 61.0% in year 2. The other two interactions, years of residence interacting with age and *Toxoplasma* IgG (*χ* ^2^_4_ = 9.603, *P* = 0.048) and years of residence interacting with nationality and *Toxoplasma* IgG (*χ* ^2^_4_ = 9.628, *P* = 0.047), were only marginally significant and thus were not explored further.

Models in which extrinsic factors were fitted with seropositivity of anti-*Toxoplasma* IgM, either alone or in combination with IgG did not identify any of factors as statistically significantly (Table 2).

## Discussion

This study investigated the status of *T. gondii* infection among migrant workers in Malaysia using standard commercial kits that detect anti-*Toxoplasma* IgG and IgM antibodies. The results showed that more than half of the workers had latent infection (53.0%), indicative of previous exposure to *T. gondi.* The high prevalence of latent *T. gondii* infection among these workers suggests that most of these infections were probably acquired in their home countries, where toxoplasmosis is known to be prevalent [27–29].

This study is the first to report on seroprevalence of *T.gondii* infections in migrant workers from multiple occupation sectors, unlike previous studies which have only reported on a single working sector at a time, e.g. the plantation sector [21–24]. Seroprevalence in our study was marginally higher (57.4%) compared to previously reported values from migrant workers in Malaysia of between 5.2 and 46.2% [21–24]. This high seroprevalence is not surprising as human infection is widely reported, with nearly one-third of the world population exposed to this parasite [2, 30, 31]. Toxoplasmosis is not exclusive to marginalized communities, but it may have a greater impact on such communities for the reasons argued below. Studies in Malaysia have reported infections (seroprevalence) in healthy individuals (13.9–30.2%) [32–34], pregnant women (23.0–31.6%) [35–38], HIV patients (21.0–41.2%) [20, 39–41], newborn babies (2.0%) [42] and indigenous communities (10.6–37.0%) [43, 44]. In Southeast Asia, seroprevalence varies from < 2% up to 70% [45]. In high income countries, such as the USA and the UK, it has been estimated that between 10 and 40% of people are infected [46–48], while in Central and South America and continental Europe prevalence ranges from 50 to 80% [49].

In the present study, two of the factors considered as intrinsic to the sampled individuals showed highly significant associations with *T. gondii* infection. The first variable was age class, with prevalence being higher among workers older than 45 years (74.3 to 76.9%) compared with younger workers (51.4 to 59.2%). This is in agreement with previous studies [44, 50–53] where infections acquired increased with age [32, 39]. A recent study among the indigenous communities of Malaysia (Orang Asli) showed significantly higher seroprevalence (*P ≤* 0.001) among those aged 12 years and older (52.6%), compared to younger participants (31.2%) [44]. In the current study, prevalence was very similar in both sexes.

The second significant factor affecting seroprevalence was the migrant workers’ countries of origin and thought to be related to behavioural and cultural practices such as unintentional ingestion of oocysts shed in cats’ faeces and/or consumption of undercooked or raw contaminated meat [44, 54, 55]. Seroprevalence (by IgG) was highest among workers from Nepal (77.8%), followed by Indonesia (58.3%), Bangladesh (45.8%), India (38.5%) and Myanmar (28.6%), in agreement with a study in 2008 [24]. The strength of country of origin as a significant explanatory factor of *T. gondii* seroprevalence is most likely due to a combination of dietary habits, behavioural risks, environmental conditions, socioeconomic status and poor personal hygiene practice [24]. High prevalence of infection is common among ethnic groups in Nepal due to their habitual ingestion of minced raw meat or insufficiently cooked meat, both of which may harbour tissue cysts of the parasite [28, 29]. Similarly, in Indonesia, *T. gondi* infection is also considered to be a food-borne disease. Gandahusada (1991) [27] linked infection in Indonesia to the presence of domestic animals and eating raw or partially cooked meat with seroprevalence ranging between 2–63% in humans, between 35–73% in cats, 75% in dogs, between 11–36% in pigs, between 11–61% in goats, and less than 10% in cows. In the present study, all the workers originated from rural areas in their respective countries where infections are highly prevalent especially among poor and deprived communities. In such communities, domestic and feral cats are the most likely sources of environmental contamination, leading to direct infections in humans or indirectly through tissue-cyst bearing domestic animals. Significant correlations between consumption of unboiled water and *T. gondii* seropositivity have also been noted in a few studies, particularly among disadvantaged and indigenous communities living in rural and remote areas, with toxoplasmosis being considered a water-borne disease in these places [14, 56–58]. Contamination of water reservoirs with cat faeces [56] and collection of water from shallow wells located on farms where infected cats are present [57, 58] constitute possible sources of human infection with *T. gondii* oocysts.

We found that infections were significantly higher among workers from the manufacturing sector (76.3%) compared to workers in other sectors. Rai et al. [29] highlighted that the nature of one’s occupation increases the risk of acquiring *T. gondii* infection especially for those engaged in agricultural activities. However, the present analysis revealed the lowest prevalence of infection (45.1%) amongst plantation workers. This latter result may be biased to some extent as working sectors were commonly dominated by a particular nationality. In Malaysia, Nepalese workers dominated the manufacturing sector (81.7% were Nepalese) and the high prevalence of infection in the manufacturing sector (74.2%) was largely attributable to the Nepalese (74.7%).

Workers with an employment history of less than one year or newly arrived workers were most frequently infected with *T. gondii* except for those in the domestic sector, indicating that acquisition of infection for most immigrant workers was most likely to be from their country of origin [27–29]. The reason for the increase in prevalence between year 1 and 2 among those in the domestic sector is not clear, but may be related to closer contact with domestic cats, which are commonly kept as pets in Malaysia. The majority of domestic workers in this study lived in their own or rented houses (98.1%) with pets and/or likely to clean cat litter trays as part of their domestic duties. By contrast, workers in other employment sectors mostly live in hostels where they are not allowed to keep pets.

Toxoplasmosis has been associated with the incidence (and prevalence) of schizophrenia and other human affective and psychotic disorders. Antipsychotic drugs known to be effective in the treatment of schizophrenia also inhibit some parasites, predominantly *T. gondii* [13, 59]. However, access to health care in general and mental health care in particular, is likely to be very low among poor and marginalized communities, with migrant workers less likely to seek care due to stigmatization and fear of job loss.

## Conclusions

In conclusion, high seroprevalence of *T. gondii* among migrant workers in Peninsular Malaysia was found to be positively and statistically significantly associated with age (> 45 years), nationality (Nepalese), employment sector (manufacturing) and shorter duration of residence in Malaysia (with the exception of domestic workers, in whom the converse association was shown). Therefore, our results call for the public health authorities in Malaysia to include a health education programme not only specifically for migrant workers (who have pivotally fueled the Malaysian economy) but also among the general public. This will help to increase public awareness of toxoplasmosis and especially about the importance of cats and the consumption of contaminated meat and water as major potential sources of infection [14, 15].

## References

[1] Feldman HA (1974). Toxoplasmosis: An overview. Bull NY Acad Med.

[2] Montoya JG, Liesenfeld O (2004). Toxoplasmosis. Lancet.

[3] Hill DE, Chirukandoth S, Dubey JP (2005). Biology and epidemiology of *Toxoplasma gondii* in man and animals. Anim Health Res Rev.

[4] Jacobs LJ (1974). *Toxoplasma gondii*: Parasitology and transmission. Bull NY Acad Med.

[5] Abu Bakar A, Khairul Anuar A (1992). Mat Ludin, Rahmah N. Survey of meat samples for *Toxoplasma gondii* in Northern Peninsular Malaysia. Diagnosa.

[6] Kolbekova’ P, Kourbatova E, Novotna’ M, Kodym P, Flegr J (2007). New and old risk-factors for *Toxoplasma gondii* infection: prospective cross-sectional study among military personnel in the Czech Republic. Clin Microbiol Infect.

[7] Ferguson DJ (2009). Identification of faecal transmission of *Toxoplasma gondii*: Small science, large characters. Int J Parasitol.

[8] Jones JL, Dargelas V, Roberts J, Press C, Remington JS, Montoya JG (2009). Risk factors for *Toxoplasma gondii* infection in the United States. Clin Infect Dis.

[9] Petersen E, Vesco G, Villari S, Buffolano W (2010). What do we know about risk factors for infection in humans with *Toxoplasma gondii* and how can we prevent infections?. Zoonoses Public Hlth.

[10] Husni OM, Bowman DD, Khairul Anuar A, Rahmah N (1994). Evaluation of strategies to reduce the risk of congenital toxoplasmosis. J Eukaryot Microbiol.

[11] Remington JS, Klein JO. Infectious diseases of the fetus and newborn infant. Philadelphia: Saunders. Xiv; 2001

[12] Hanifa, Khairul Anuar A, Rahmah N (1996). Cerebral toxoplasmosis in AIDS: A case report and literature review. Trop Biomed.

[13] Yolken RH, Dickerson FB, Torrey EF (2009). Toxoplasma and schizophrenia. Parasite Immunol.

[14] Torrey EF, Yolken RH (2013). *Toxoplasma* oocysts as a public health problem. Trends Parasitol.

[15] Torrey EF, Simmons W, Yolken RH (2015). Is childhood cat ownership a risk factor for schizophrenia later in life?. Schizophr Res.

[16] Alvarado-Esquivel C, Sifuentes-Álvarez A, Narro-Duarte SG, Estrada-Martínez S, Díaz-García JH, Liesenfeld O (2006). Seroepidemiology of *Toxoplasma gondii* infection in pregnant women in a public hospital in northern Mexico. BMC Infect Dis.

[17] Binnicker MJ, Jespersen DJ, Harring JA (2010). Multiplex detection of IgM and IgG class antibodies to *Toxoplasma gondii*, rubella virus, and cytomegalovirus using a novel multiplex floww immunoassay. Clin Vaccine Immunol.

[18] Mwambe B, Mshana SE, Kidenya BR, Massinde AN, Mazigo HD, Micheal D (2013). Sero-prevalence and factors associated with *Toxoplasma gondii* infection among pregnant women attending antenatal care in Mwanza. Tanzania Parasit Vectors.

[19] Yahaya N (1991). Review of toxoplasmosis in Malaysia. Southeast Asian J Trop Med Public Health.

[20] Nissapatorn V, Kamarulzaman A, Init I, Tan LH, Rohela M, Norliza A (2002). Seroepidemiology of toxoplasmosis among HIV infected patients and healthy blood donors. Med J Malaysia.

[21] Chan BTE, Amal RN, Noor Hayati MI (2009). Toxoplasmosis among Indonesian Migrant Workers in Malaysia. Malays J Med Sci.

[22] Chan BTE, Amal RN, Noor Hayati MI, Hideto K, Anisah N, Norhayati M (2009). Comparative study of seroprevalence of toxoplasmosis between local workers and migrant workers in Malaysia. Arch Med Sci.

[23] Amal RN, Chan BTE, Noor Hayati MI, Kino H, Anisah N, Norhayati M (2008). Seroprevalence of toxoplasmosis: comparative study between migrant workers from the Indian subcontinent and local Malaysian workers. Iraqi J Med.

[24] Chan BTE, Amal RN, Noor Hayati MI, Kino H, Anisah N, Norhayati M (2008). Seroprevalence of toxoplasmosis among migrant workers from different Asian countries working in Malaysia. Southeast Asian J Trop Med Public Health.

[25] Norhayati M, Fatmah MS, Yusof S, Edariah AB (2003). Intestinal parasitic infections in man: a review. Med J Malaysia.

[26] Rohlf FJ, Sokal RR (1995). Statistical Tables 3rd edition.

[27] Gandahusada S (1991). Study on the prevalence of toxoplasmosis in Indonesia: a review. Southeast Asian J Trop Med Public Health.

[28] Rai SK, Shibata H, Sumi K, Kubota K, Hirai K, Matsuoka A (1994). Seroepidemiological study of toxoplasmosis in two different geographical areas in Nepal. Southeast Asian J Trop Med Public Health.

[29] Rai SK, Matsumura T, Ono K, Abe A, Hirai K (1999). High *Toxoplasma* seroprevalence associated with meat eating habits of locals in Nepal. Asia Pac J Public Health.

[30] Dubey JP, Beattie CP (1988). Toxoplasmosis of animals and man.

[31] Dubey JP (2010). Toxoplasmosis of animals and humans.

[32] Tan DSK, Zaman V (1973). *Toxoplasma* antibody survey in West Malaysia. Med J Malaysia.

[33] Thomas V, Sinniah B, Yap PL (1980). Prevalence of antibodies including IgM to *Toxoplasma gondii* in Malaysia. Southeast Asian J Trop Med Public Health.

[34] Sinniah B, Thomas V, Yap PL (1984). Toxoplasmosis in West Malaysian population. Trop Biomed.

[35] Cheah WC, Fah CS, Fook CW (1975). Pattern of toxoplasma antibodies in Malaysian pregnant women. Med J Malaya.

[36] Tan SDK, Cheah W, Sukumaran KD, Stern H (1976). The “TORCHES” (congenital toxoplasmosis) programme 1 in women of child-bearing age. Singapore Med J.

[37] Khairul Anuar A, Afifi AB, Dighe VC, Rosiani AMM (1991). *Toxoplasma* antibody in pregnant women in northern Peninsular Malaysia. Diagnosis.

[38] Ravichandran J, Rahmah N, Kamaruzzaman A, Khairul Anuar A (1998). *Toxoplasma gondii* antibodies among Malaysian pregnant women: A hospital-based study. Biomed Res.

[39] Nissapatorn V, Lee CKC, Cho SM, Rohela M, Khairul Anuar A (2003). Toxoplasmosis in HIV/AIDS patients in Malaysia. Southeast Asian J Trop Med Public Health.

[40] Nissapatorn V, Lee CKC, Khairul AA (2003). Seroprevalence of toxoplasmosis among AIDS patients in Hospital Kuala Lumpur, 2001. Singapore Med J.

[41] Nissapatorn V, Lee C, Quek KF, Khairul AA (2003). AIDS-related opportunistic infections in Hospital Kuala Lumpur. Jpn J Infect Dis.

[42] Tan DSK, Mak JW (1985). The role of toxoplasmosis in congenital disease in Malaysia. Southeast Asian J Trop Med Public Health.

[43] Lokman HS, Radzan T, Nazma M (1994). Distribution of anti-*Toxoplasma gondii* antibodies among Orang Asli (Aborigines) in peninsular Malaysia. Southeast Asian J Trop Med Public Health.

[44] Ngui R, Lim YAL, Amir NFH, Nissapatorn V, Mahmud R (2011). Seroprevalence and sources of toxoplasmosis among Orang Asli (indigenous) communities in Peninsular Malaysia. Am J Trop Med Hyg.

[45] Nissapatorn V (2007). Toxoplasmosis: A silent threat in Southeast Asia. Res J Parasitol.

[46] Bhatia VN, Meenakshi K, Aggarwal SC (1974). Toxoplasmosis in South India, a serological study. Indian J Med Res.

[47] Dubey JP (1994). Toxoplasmosis. J Am Vet Med Assoc.

[48] Sukthana Y (2006). Toxoplasmosis: beyond animals to humans. Trends Parasitol.

[49] Jones JL, Kruszon-Moran D, Wilson M (2007). *Toxoplasma gondii* prevalence, United States [letter]. Emerg Infect Dis.

[50] Tenter AM, Heckeroth AR, Weiss LM (2000). *Toxoplasma gondii*: from animals to humans. Int J Parasitol.

[51] Nissapatorn V, Abdullah KA (2004). Review on human toxoplasmosis in Malaysia: the past, present and prospective future. Southeast Asian J Trop Med Public Health.

[52] Sobral CA, Amendoeira MR, Teva A, Patel BN, Klein CH (2005). Seroprevalence of infection with *Toxoplasma gondii* in indigenous Brazilian populations. Am J Trop Med Hyg.

[53] Abu-Madi MA, Al-Molawi N, Behnke JM (2008). Seroprevalence and epidemiological correlates of *Toxoplasma gondii* infections among patients referred for hospital-based serological testing in Doha, Qatar. Parasit Vectors.

[54] Apt W, Thiermann E, Niedmann G, Pasmanik S (1973). Toxoplasmosis.

[55] Amendoeira MRR, Costa T, Spalding SM (1999). *Toxoplasma gondii* Nicolle & Manceaux, 1909 (Apicomplexa: Sarcocystidae) e atoxoplasmose. Revista Souza Marques.

[56] de Moura L, Bahia-Oliveira LM, Wada MY, Jones JL, Tuboi SH, Carmo EH (2006). Waterborne toxoplasmosis, Brazil, from field to gene. Emerg Infect Dis.

[57] Sroka J, Wójcik-Fatla A, Dutkiewicz J (2006). Occurrence of *Toxoplasma gondii* in water from wells located on farms. Ann Agric Environ Med.

[58] Sroka J, Wojcik-Fatla A, Szymanska J, Dutkiewicz J, Zajac V, Zwolinski J. The occurrence of *Toxoplasma gondii* infection in people and animals from rural environment of Lublin region - estimate of potential role of water as a source of infection. Ann Agric Environ Med. 2010;17:125–32. 20684490

[59] Webster JP, Lamberton PHL, Donnelly CA, Torrey EF (2006). Parasites as causative agents of human affective disorders? The impact of anti-psychotic, mood-stabilizer and anti-parasite medication on *Toxoplasma gondii*’s ability to alter host behaviour. Proc R Soc B (Biol Sci).

